# Minimum clinical important difference for resilience scale specific to cancer: a prospective analysis

**DOI:** 10.1186/s12955-020-01631-6

**Published:** 2020-12-09

**Authors:** Zeng Jie Ye, Zhang Zhang, Ying Tang, Jian Liang, Xiao Ying Zhang, Guang Yun Hu, Zhe Sun, Mu Zi Liang, Yuan Liang Yu

**Affiliations:** 1 Guangzhou University of Chinese Medicine, Guangzhou, 510006 Guangdong Province China; 2 Sun Yat-sen University Cancer Center, State Key Laboratory of Oncology in South China, Collaborative Innovation Center for Cancer Medicine, Guangzhou, 510060 Guangdong Province China; 3 Institute of Tumor, Guangzhou University of Chinese Medicine, Guangzhou, 510006 Guangdong Province China; 4 Guangdong Provincial Key Laboratory of New Drug Development and Research of Chinese Medicine, Mathematical Engineering Academy of Chinese Medicine, Guangzhou University of Chinese Medicine, Guangzhou, 510006 Guangdong Province China; 5 The Seventh Affiliated Hospital, Sun Yat-sen University, Shenzhen, 510275 Guangdong Province China; 6 Army Medical University, Chongqing Municipality, 400038 China; 7 The First Affiliated Hospital, Guangzhou University of Chinese Medicine, Guangzhou, 510405 Guangdong Province China; 8 Guangdong Academy of Population Development, Guangzhou, 510600 Guangdong Province China; 9 South China University of Technology, Guangzhou, 510641 Guangdong Province China

**Keywords:** Resilience, Nursing, Cancer, RS-SC-25, RS-SC-10, Minimum clinical important difference, Anchor-based, Distribution-based, ROC

## Abstract

**Background:**

The minimum clinical important differences (MCIDs) of resilience instruments in patients with cancer have not been comprehensively described. This study was designed to evaluate MCIDs of 10-item and 25-item resilience scales specific to cancer (RS-SC-10 and RS-SC-25).

**Methods:**

From June 2015 to December 2018, RS-SCs were longitudinally measured in 765 patients with different cancer diagnoses at baseline (T0) and 3 months later (T1). The EORTC QLQ-C30, Connor-Davidson Resilience Scale, Hospital Anxiety and Depression Scale, and Allostatic Load Index were measured concurrently as anchors. Anchor-based methods (linear regression, within-group), distribution-based methods(within-group), and receiver operating characteristic curves (ROCs, within-subject) were performed to evaluate the MCIDs.

**Results:**

623 of 765 (84.1%) patients had paired RS-SCs scores. Moderate correlations were identified between the change in RS-SCs and change in anchors (*r* = 0.38–0.44, all *p* < 0.001). Linear regression estimated + 8.9 and − 6.7 as the MCIDs of RS-SC-25, and + 3.4 and − 2.5 for RS-SC-10. Distribution-based methods estimated + 9.9 and − 9.9 as the MCIDs of RS-SC-25, and + 4.0 and − 4.0 for RS-SC-10. ROC estimated + 5.5 and − 4.5 as the MCIDs of RS-SC-25, and + 2.0 and − 1.5 for RS-SC-10.

**Conclusions:**

The most reliable MCID is around 5 points for RS-SC-25 and 2 points for RS-SC-10. RS-SCs are more responsive to the worsening status of resilience in patients with cancer and these estimates could be useful in future resilience-based intervention trials.

## Introduction

In China, about 4.5 million people were diagnosed with cancer and 2.9 million people died from it in 2019 [1]. The mortality of cancer has been lowered owing to advances in medical technology, and cancer is increasingly treated as a chronic disease [2]. Thus, helping cancer survivors rehabilitate from the traumatic event is a topic receiving enhanced interest in cancer research. Resilience is defined as one’s ability to ‘bounce back’ from adversity and is a salient indicator of patients’ quality of life and psychosocial functions [3, 4]. However, no resilience scales have been formulated to be specific to patients with cancer, and the application of generic resilience instruments among cancer-specific populations has been criticized [5]. To address this issue, we developed a 25-item Resilience Scale Specific to Cancer (RS-SC-25) as an aid for nurses, physicians and social workers to assess the resilience levels of patients [6]; RS-SC-25 was then validated based on classic theory test, item response theory, and resilience-related empirical research [7, 8]. The RS-SC-25 has Chinese and English versions and consists of five domains (generic element, benefit finding, support and coping, hope for the future, and meaning for existence); higher scores indicate higher resilience levels [6]. RS-SC-25 has good psychometric properties and could be used as a means of establishing a symptomatic threshold to guide the initiation of psychosocial or pharmacological intervention. A short 10-item version (RS-SC-10) with higher discriminative items has been developed according to multidimensional item response theory (MIRT) to reduce the scale burden on the patients; its potential in outpatient wards and communities has been confirmed [9]. However, the responsiveness of both RS-SCs (RS-SC-25 and RS-SC-10), that is, its ability to measure changes over time, has not been evaluated, raising concerns on the use of this instrument in routine practice. To enable an appropriate assessment of resilience-related changes in patients with cancer, the minimum clinically important difference (MCID), which is the smallest change in score that patients perceive as beneficial or detrimental, is important to the clinical interpretation of scale data, especially for resilience-related interventions in different settings [10, 11]. However, to our knowledge, the MCID of resilience instruments in patients with cancer have not been comprehensively described. Thus, the current study was designed to calculate the MCIDs of RS-SCs using distribution- and anchor-based methods. Based on previous findings [4, 6–8, 12–14], resilience was strongly associated with anxiety, depression, quality of life, and Allostatic Load Index. Thus, we postulated the following: (1) RS-SCs would be significantly associated with established scales, namely, European Organization for Research and Treatment of Cancer Quality of Life Questionnaire (EORTC QLQ-C30, measuring quality of life), Connor-Davidson Resilience Scale (CD-RISC, measuring generic resilience), Hospital Anxiety and Depression Scale (HADS, measuring anxiety and depression), and Allostatic Load Index (ALI, measuring physiological load) in this study; and (2) changes in RS-SCs would be significantly associated with changes in established scales.

## Methods

### Sample/participants

Participants were enrolled from six hospitals in Guangdong and Heilongjiang Provinces between June 2015 and December 2018; each had a confirmed diagnosis of cancer based on biopsy and medical imaging. All participants had to fulfill the same inclusion/exclusion criteria, which were detailed as follows, inclusion: (1) aged 18–65 years, (2) had the ability to communicate in Mandarin or Cantonese fluently, and (3) receiving active treatment; exclusion: (1) misdiagnosed with cancer, (2) cannot communicate in Mandarin or Cantonese fluently, and (3) unwilling to participate in the study. Patients were all derived from a big project named as Be Resilient to Cancer. Informed consent was obtained, and the Human Research Ethics Committee approved the present study (registration number: 2016KYTD08).

### Data collection

This prospective study was conducted between June 2015 and December 2018. RS-SCs, EORTC QLQ-C30, CD-RISC, and HADS were administered to 765 patients at baseline (T0), whereas ALI was conducted in 275 patients treated in the six hospitals. Three months later (T1), the same instruments were administered again.

### Measures

#### RS-SC

The original RS-SC is a 25-item resilience instrument specific to cancer (RS-SC-25) that has the five domains of generic element, benefit finding, support and coping, hope for the future, and meaning for existence [6]. A 10-item RS-SC (RS-SC-10) was developed later by MIRT analysis [9]. The two scales are both rated based on a five-point Likert scale, with higher scores indicating higher resilience levels. Scores for RS-SC-25 range from 25 to 125, and for RS-SC-10, from 10 to 50. The Cronbach’s α and test-retest reliability of RS-SC-25 are 0.83 and 0.87, respectively [6, 9, 15]. The Cronbach’s α of RS-SC-10 is 0.86. RS-SC-25 and RS-SC-10 are attached in the Additional file 1 (Additional file 1: Tables S1 and S2).

### EORTC QLQ-C30

EORTC QLQ-C30 is a 30-item quality of life (QoL) instrument specific to cancer, including five functional dimensions, three symptom dimensions, a global health status, some additional symptom items reported by patients with cancer, and perceived financial impact of cancer [16]. The raw score of each dimension can be converted to a score ranging from 0 to 100 according to the manual, with higher scores indicating better functional ability or increased distress in the symptom items [17]. In addition, Item 30 measuring the global health status was used as an anchor in this study (raw score ranging from 1 to 7). The Cronbach’s α of the Chinese version of EORTC QLQ-C30 ranges from 0.78 to 0.93 in different dimensions.

### CD-RISC

The Chinese version of CD-RISC is a 25-item generic resilience instrument with the three dimensions of tenacity, strength, and optimism [18]. Additional two short non-dimensional versions of CD-RISC (2- and 10-item, named CD-RISC-2 and CD-RISC-10, respectively) were developed later. These three scales are all rated based on a five-point Likert scale with higher scores indicating higher resilience levels (ranging from 0 to 8, 0–40, 0–100, respectively). CD-RISC-10 was used as an anchor in this study. Cronbach’s αs of 0.83, 0.79, and 0.85 are identified for CD-RISC-25, CD-RISC-2, and CD-RISC-10, respectively [19, 20].

### HADS

The Chinese version of HADS is a 14-item emotional distress-screening tool with seven items for anxiety and seven items for depression [21]. The instrument is scored on a five-point scale with higher scores indicating worse emotional functions (ranging from 0 to 56). HADS was used as an anchor in this study. The Cronbach’s α of HADS is 0.91.

### ALI

ALI is a validated composite index measuring 14 indicators from different physiological systems, such as the functions of the sympathetic nervous system, parasympathetic nervous system, and hypothalamic pituitary adrenal. ALI scores range from 0 to 14, with higher scores indicating higher allostatic load. Similar approaches to conceptualize physical allostatic load have been applied [22–25]. The Cronbach’s α of ALI has not been evaluated.

### Data analysis

First, at T0, Pearson’s r correlation coefficients were used to measure the correlations between RS-SCs (RS-SC-25 and RS-SC-10) and anchors in patients with different cancer diagnoses [26, 27]. Fisher’s z-transformation was applied to approximate the variance-stabilizing transformation for Pearson’s r correlation coefficients when RS-SCs and anchors followed a bivariate normal distribution, and determine the 95% confidence intervals (CIs) for the Pearson’s r correlation coefficients [28, 29]. r < 0.3, 0.3 ≤ r ≤ 0.5, r > 0.5 were defined as weak, moderate and strong coefficients, respectively.

Second, from T0 to T1, Pearson’s r correlation coefficients (95%CI) were performed again to estimate the correlations between change in RS-SCs and change in anchors, in order to select suitable anchors. The correlation coefficients should be more than 0.30, as recommended [11].

Third, from T0 to T1, linear regression (95%CI, within-group) were calculated to compare changes in RS-SCs against different anchors. As for linear regression, change in RS-SC score (independent variable) was anchored against change in EORTC QLQ-C30, CD-RISC, and HADS score (dependent variables), respectively. In addition, Cohen effect size (ES) was also calculated [30]. ES < 0.3, 0.3 ≤ ES ≤ 0.8, ES > 0.8 were defined as small, medium, and large effect. For the distribution-based estimation (within-group) of MCIDs, we calculated the 20% (0.2 SD), 30% (0.3 SD), and 50% (0.5 SD) SD [31]; standard error of measurement (SEM) [32]; and minimal detectable change (MDC) for the 90%CI (MDC_90_) and 95%CI (MDC_95_) [10].

At last, from T0 to T1, receiver operating characteristic curves (ROCs, within-subject) analysis were performed. Changes in the cut-off of RS-SCs that best discriminated between patients who increased or decreased their resilience levels by the established MCIDs in the EORTC QLQ-C30 (1-point change in global health status, QoL-GHS) [33], CD-RISC-10 (3-point change) [34], and HADS (1.5-point change for anxiety and depression each) were defined as showing MCID [35]. The area under curve (AUC) and Youden index were adapted with equal weighting given to sensitivity and specificity [36]. *P* < 0.05 was recognized as statistically significant for all the data analysis. All data analyses were performed using SPSS 21 (IBM, USA).

## Results

### Demographics

623 of 765 (84.1%) patients had paired RS-SCs scores. Breast, gastric, and lung cancer were the three most common cancer diagnoses, constituting 33.5, 20.1,13.3% of all cases, respectively. One hundred forty-two patients were excluded from the analysis for the following reasons:unwillingness (*N* = 37), busy schedule (*N* = 24), lost to follow-up (*N* = 55), and incomplete responses (*N* = 26). No significant demographic difference was identified between the included and the excluded. The baseline characteristics of the patients are presented in Table 1.

**Table 1:** Characteristics of patients in the analysis of RS-SCs (*N* = 765)

Characteristics (%)	Lung cancer	Gastric cancer	Colon-rectal cancer	Liver cancer	Leukemia	Breast cancer
No	102 (13.3)	154 (20.1)	98 (12.8)	81 (10.6)	74 (9.7)	256 (33.5)
Sex						
Female	29 (28.4)	73 (47.4)	50 (51.0)	29 (35.8)	34 (45.9)	256 (100.0)
Man	73 (71.6)	81 (52.6)	48 (49.0)	52 (64.2)	40 (54.1)	0 (0.0)
Age (years, SD)	44.7 (17.1)	57.1 (16.9)	53.1 (14.8)	46.8 (13.9)	39.1 (16.9)	39.3 (14.7)
Time since first confirmed diagnosis (months, SD)	14.1 (12.0)	17.8 (8.7)	20.9 (12.5)	9.9 (10.1)	8.1 (6.9)	5.2 (6.1)
Duration of treatment (months, SD)	6.7 (5.5)	11.9 (8.0)	15.9 (9.2)	6.3 (5.4)	7.1 (8.0)	4.6 (7.4)
Education level						
Middle school or lower	58 (56.9)	91 (59.1)	52 (53.1)	42 (51.9)	43 (58.1)	112 (43.8)
High school or higher	44 (43.1)	63 (40.9)	46 (46.9)	39 (48.1)	31 (41.9)	144 (56.2)
Family income (RMB per month)						
< 5000	47 (46.1)	85 (55.2)	49 (50.0)	39 (48.1)	34 (45.9)	97 (37.9)
5000–10,000	28 (27.5)	41 (26.6)	26 (26.5)	32 (39.5)	28 (37.8)	116 (45.3)
> 10,000	27 (26.4)	28 (18.2)	23 (23.5)	10 (12.4)	12 (16.2)	43 (16.8)
Marital status						
Married	85 (83.3)	124 (80.5)	77 (78.6)	62 (76.5)	58 (78.4)	157 (61.3)
Single or other	17 (16.7)	30 (19.5)	21 (21.4)	19 (23.5)	16 (21.6)	99 (38.7)
Religious beliefs						
Yes	18 (17.6)	41 (26.6)	28 (28.6)	28 (34.6)	21 (28.4)	65 (25.4)
None	84 (82.4)	113 (73.4)	70 (71.4)	53 (65.4)	53 (71.6)	191 (74.6)
Employment status						
Employment	54 (52.9)	76 (49.4)	43 (43.9)	30 (37.0)	38 (51.4)	162 (63.3)
Unemployment	48 (47.1)	78 (50.6)	55 (56.1)	51 (63.0)	36 (48.6)	94 (36.7)
Stage of cancer						
I	15 (14.7)	19 (12.3)	21 (21.4)	15 (18.5)	NA	77 (30.1)
II	29 (28.4)	71 (46.1)	23 (23.5)	28 (34.6)	NA	87 (34.0)
III	41 (40.2)	46 (29.9)	32 (32.7)	25 (30.9)	NA	74 (28.9)
IV	17 (16.7)	18 (11.7)	22 (22.4)	13 (16.0)	NA	18 (7.0)
Type of therapy						
Chemo	41 (40.2)	63 (40.9)	45 (45.9)	57 (70.4)	56 (75.7)	146 (57.0)
Radiation	23 (22.5)	32 (20.8)	30 (30.6)	31 (38.3)	13 (17.6)	54 (21.1)
Surgery	22 (21.6)	84 (54.5)	57 (58.2)	37 (45.7)	10 (13.5)	138 (53.9)
Combordities						
None	25 (34.3)	55 (35.7)	26 (26.5)	25 (30.9)	40 (54.1)	152 (59.4)
One	36 (35.3)	62 (40.3)	41 (41.8)	38 (46.9)	24 (32.4)	83 (32.4)
Two or more	41 (40.2)	37 (24.0)	31 (31.7)	18 (22.2)	10 (13.5)	21 (8.2)

*NA* not available

### Correlations between RS-SCs and anchors at baseline (T0)

The means (SD) of the RS-SCs and anchors are described in Table 2. Acceptable correlations (from 0.38 to 0.73, *P* < 0.001) were identified between RS-SCs and anchors. In general, the correlations between RS-SC-10 and established scales were stronger than those between RS-SC-25 and external indicators.

**Table 2:** Pearson’s correlation coefficients between RS-SCs and potential anchors at baseline (T_0_, *N* = 765)

Variables	Mean (SD)	Score range	Item No	Cronbach α	RS-SC-10	RS-SC-25		
					r (95%CI)	P	r (95%CI)	P
QoL GHS	55.73 (20.62)	0–100	1	NA	0.54 (0.48 to 0.59)	< 0.001	0.51 (0.45 to 0.57)	< 0.001
QoL PF	64.86 (29.37)	0–100	5	0.85	0.39 (0.32 to 0.45)	< 0.001	0.38 (0.31 to 0.44)	< 0.001
QoL RF	47.89 (19.89)	0–100	2	0.79	0.56 (0.50 to 0.61)	< 0.001	0.53 (0.47 to 0.58)	< 0.001
QoL EF	40.54 (16.91)	0–100	4	0.86	0.65 (0.60 to 0.69)	< 0.001	0.60 (0.55 to 0.65)	< 0.001
QoL CF	58.97 (21.12)	0–100	2	0.74	0.53 (0.47 to 0.58)	< 0.001	0.52 (0.46 to 0.57)	< 0.001
QoL SF	68.78 (30.66)	0–100	2	0.76	0.61 (0.56 to 0.66)	< 0.001	0.57 (0.51 to 0.62)	< 0.001
CD-RISC-25	46.34 (21.88)	0–100	25	0.83	0.64 (0.59 to 0.68)	< 0.001	0.59 (0.54 to 0.64)	< 0.001
CD-RISC-10	18.85 (6.93)	0–40	10	0.85	0.73 (0.69 to 0.76)	< 0.001	0.67 (0.62 to 0.71)	< 0.001
CD-RISC-2	3.48 (1.46)	0–8	2	0.74	0.51 (0.45 to 0.57)	< 0.001	0.48 (0.42 to 0.54)	< 0.001
HADS total	18.08 (6.95)	0–42	14	0.80	− 0.59 (− 0.64 to − 0.54)	< 0.001	− 0.56 (− 0.61 to − 0.50)	< 0.001
HADS-A	8.43 (3.12)	0–21	7	0.84	− 0.46 (− 0.52 to − 0.40)	< 0.001	− 0.45 (− 0.51 to − 0.39)	< 0.001
HADS-D	9.65 (3.89)	0–21	7	0.87	− 0.68 (− 0.72 to − 0.64)	< 0.001	− 0.59 (− 0.64 to − 0.54)	< 0.001
ALI	5.86 (2.87)	0–14	14	NA	− 0.45 (− 0.51 to − 0.39)	< 0.001	− 0.43 (− 0.49 to − 0.36)	< 0.001

*RS-SC* Resilience Scale Specific to Cancer, *HADS* Hospital Anxiety and Depression Scale, *QoL* Quality of Life, *GHS* Global Health Status, *PF* Physical Function, *RF* Role Function, *EF* Emotion Function, *CF* Cognitive Function, *SF* Social Function, *CD-RISC* Connor-Davidson Resilience Scale, *ALI* Allostatic Load Index, *NA* not available

### Correlations between changes in RS-SCs and changes in anchors from T0 to T1

The correlations between change in RS-SCs and change in anchors are presented in Table 3. Changes in QoL-GHS (*r* = 0.38(0.28 to 0.47), ES = 0.26, *P* < 0.001), CD-RISC-10 (0.44(0.35 to 0.52), ES = 0.21, *P* < 0.001), HADS-A (*r* = − 0.35(− 0.44 to − 0.25), ES = 0.19, *P* < 0.001), HADS-D (*r* = −-0.41(− 0.50 to − 0.31), ES = 0.23, *P* < 0.001), significantly correlated with changes in RS-SC-25 except for ALI (*r* = 0.19(− 0.29 to − 0.08), ES = 0.11, *P* = 0.001). Similar results were identified between RS-SC-10 and anchors. Thus, ALI could not be used as an anchor in the MCID analysis.

**Table 3:** Pearson’s correlations between change in RS-SCs and change in potential anchors at 3-month (T_1_, *N* = 643)

Variables	Changes (95%CI)	Effect size	RS-SC-10		RS-SC-25	
			r (95%CI)	P	r (95%CI)	P
QoL GHS	*5.13 (3.34 to 7.20)*	*0.26*	*0.38 (0.28 to 0.47)*	< *0.001*	*0.32 (0.22 to 0.41)*	< *0.001*
QoL PF	1.21 (0.96 to 1.51)	0.05	0.15 (0.04 to 0.26)	0.092	0.18 (0.08 to 0.28)	0.004
QoL RF	2.52 (2.30–2.79)	0.16	0.31 (0.21 to 0.41)	< 0.001	0.26 (0.16 to 0.35)	< 0.001
QoL EF	4.04 (3.63 to 4.39)	0.23	0.39 (0.29 to 0.48)	< 0.001	0.33 (0.23 to 0.42)	< 0.001
QoL CF	0.55 (0.37 to 0.74)	0.03	0.16 (0.05 to 0.27)	0.061	0.17 (0.07 to 0.27)	0.029
QoL SF	3.51 (3.19–3.81)	0.13	0.32 (0.22 to 0.42)	< 0.001	0.32 (0.22 to 0.41)	< 0.001
CD-RISC-25	6.12 (5.54 to 6.81)	0.27	0.36 (0.26 to 0.45)	< 0.001	0.34 (0.24 to 0.43)	< 0.001
CD-RISC-10	*1.22 (0.97 to 1.54)*	*0.21*	*0.44 (0.35 to 0.52)*	< *0.001*	*0.38 (0.29 to 0.47)*	< *0.001*
CD-RISC-2	0.24 (0.18 to 0.29)	0.19	0.32 (0.22 to 0.42)	< 0.001	0.29 (0.19 to 0.38)	< 0.001
HADS total	− 1.26 (− 1.45 to − 1.07)	− 0.22	− 0.38 (− 0.47 to − 0.28)	< 0.001	− 0.34 (− 0.43 to − 0.24)	< 0.001
HADS-A	− 0.51 (− 0.64 to − 0.38)	− 0.19	− 0.35 (− 0.44 to − 0.25)	< 0.001	− 0.30 (− 0.39 to − 0.20)	< 0.001
HADS− D	− 0.75 (− 0.89 to − 0.60)	− 0.23	− 0.41 (− 0.50 to − 0.31)	< 0.001	− 0.36 (− 0.45 to − 0.27)	< 0.001
ALI	− 0.27 (− 0.32 to − 0.22)	− 0.11	− 0.19 (− 0.29 to − 0.08)	0.001	− 0.20 (− 0.30 to − 0.10)	< 0.001

Italics rows indicate suitable anchors

*RS-SC* Resilience Scale Specific to Cancer, *HADS* Hospital Anxiety and Depression Scale, *QoL* Quality of Life, *GHS* Global Health Status, *PF* Physical Function, *RF* Role Function, *EF* Emotion Function, *CF* Cognitive Function, *SF* Social Function, *CD-RISC* Connor-Davidson Resilience Scale, *ALI* Allostatic Load Index

### MCID estimation of RS-SCs from T0 to T1

Using the distribution-based indicator of SEM, the mean MCIDs of RS-SC-10 and RS-SC-25 were ± 3.51 and ± 9.18, respectively. Using the distribution-based indicator of 0.5 SD, the mean MCIDs of RS-SC-10 and RS-SC-25 were ± 3.95 and ± 9.92, respectively. Other information on distribution-based indicators is given in Table 4. As for anchor-based estimates of MCID, EORTC QLQ-C30, CD-RISC, and HADS were applied as anchors owing to their strong correlation with RS-SCs (correlation coefficient > 0.3). As shown in Table 5, for patients with recovery status (improvement), linear regression estimates of the MCIDs ranged from 3.05 to 3.62 for RS-SC-10, and 8.56 to 9.31 for RS-SC-25. ROC consistently showed that a + 2.0-point change in RS-SC-10 had the best discriminant, with AUC ranging from 0.67 to 0.73, and a + 5.5-point change in RS-SC-25 had the best discriminant, with AUC ranging from 0.65 to 0.70. For patients with worsening status (deterioration), linear regression estimates of the MCID ranged from − 2.61 to − 2.26 for RS-SC-10 and ranged from − 7.05 to − 6.19 for RS-SC-25. ROC consistently showed that a − 1.5-point change in RS-SC-10 had the best discriminant, with AUC ranging from 0.66 to 0.74, and a − 4.5-point change in RS-SC-25 had the best discriminant, with AUC ranging from 0.66 to 0.73. All estimates of the MCIDs for RS-SCs are outlined in Table 6.

**Table 4:** Distribution-based MCID for RS-SCs at baseline (T_0_, *N* = 765) and 3-month (T_1_, *N* = 643)

Scales	T0						T1					
	S.E.M	MDC90	MDC95	0.2SD	0.3SD	0.5SD	S.E.M	MDC90	MDC95	0.2SD	0.3SD	0.5SD
RS-SC-10	3.28	7.66	9.10	1.70	2.54	4.24	3.74	8.72	10.35	1.81	2.72	4.53
RS-SC-25	8.01	18.70	22.21	3.78	5.67	9.45	10.35	24.15	28.68	4.75	7.12	11.87

*S.E.M* Standard Error of Measurement, *SD* Standard Deviation, *MDC* Minimal Detectable Change

**Table 5:** Anchor-based MCID and ROC estimates for RS-SC at 3-month (T_1_, *N* = 643)

Indicators	Scale	Anchors	Mean change (95%CI)	Effect Size	Cut-off	Sen	Spe	YI	AUC	P
^a^Improvement	RS-SC-10	QoL GHS	3.62 (3.34 to 3.91)	0.48	2.00	0.62	0.69	0.31	0.68	< 0.001
		CD-RISC-10	3.29 (3.10 to 3.48)	0.41	2.00	0.71	0.69	0.40	0.73	< 0.001
		HADS-A	3.05 (2.79 to 3.20)	0.40	2.00	0.61	0.66	0.27	0.67	< 0.001
		HADS-D	3.42 (3.18 to 3.66)	0.44	2.00	0.69	0.64	0.33	0.69	< 0.001
	RS-SC-25	QoL GHS	9.31 (8.83 to 9.81)	0.51	5.50	0.58	0.67	0.25	0.65	< 0.001
		CD-RISC-10	8.56 (8.23 to 8.91)	0.41	5.50	0.72	0.65	0.37	0.70	< 0.001
		HADS-A	8.78 (8.29 to 9.29)	0.44	5.50	0.59	0.67	0.26	0.66	< 0.001
		HADS-D	8.97 (8.50 to 9.44)	0.48	5.50	0.70	0.61	0.31	0.68	< 0.001
^b^Deterioration	RS-SC-10	QoL GHS	− 2.59 (− 2.79 to − 2.39)	− 0.35	− 1.50	0.64	0.68	0.32	0.69	< 0.001
		CD-RISC-10	− 2.26 (− 2.39 to − 2.14)	− 0.28	− 1.50	0.71	0.71	0.42	0.74	< 0.001
		HADS-A	− 2.61 (− 2.77 to − 2.43)	− 0.34	− 1.50	0.60	0.66	0.26	0.66	< 0.001
		HADS-D	− 2.48 (− 2.65 to − 2.32)	− 0.32	− 1.50	0.74	0.67	0.41	0.72	< 0.001
	RS-SC-25	QoL GHS	− 6.81 (− 7.18 to − 6.42)	− 0.37	− 4.50	0.61	0.68	0.29	0.68	< 0.001
		CD-RISC-10	− 6.19 (− 6.64 to − 5.93)	− 0.29	− 4.50	0.72	0.69	0.41	0.73	< 0.001
		HADS-A	− 7.05 (− 7.39 to − 6.70)	− 0.36	− 4.50	0.59	0.68	0.27	0.66	< 0.001
		HADS-D	− 6.79 (− 7.11 to 6.47)	− 0.36	− 4.50	0.72	0.64	0.36	0.70	< 0.001

*Sen* Sensitivity, *Spe* Specificity, *YI* Youden Index, *AUC* area under curve or C-statistic

^a^ Improvement was defined for QoL GHS (raw score, increased by 1 point), CD-RISC-10 (increased by 3 points), HADS-A (decreased by 1.5 points) and HADS-D (decreased by 1.5 points)

^b^ Deterioration was defined for QoL GHS (raw score, decreased by 1 point in global health status), CD-RISC-10 (decreased by 3 points), HADS-A (increased by 1.5 points) and HADS-D (increased by 1.5 points)

**Table 6:** Summary of anchor-based and distribution-based estimates for the MCID of RS-SCs

Scale	Approach	Anchor/method	MCID estimate	
			Improvement	Deterioration
RS-SC-10	Distribution	S.E.M	3.51^a^	− 3.51^a^
	Distribution	0.5SD	4.39^a^	− 4.39^a^
	Linear regression	QoL GHS	3.62	− 2.59
	Linear regression	CD-RISC-10	3.29	− 2.26
	Linear regression	HADS-A	3.05	− 2.61
	Linear regression	HADS-D	3.42	− 2.48
	ROC	QoL GHS	2.00	− 1.50
	ROC	CD-RISC-10	2.00	− 1.50
	ROC	HADS-A	2.00	− 1.50
	ROC	HADS-D	2.00	− 1.50
RS-SC-25	Distribution	S.E.M	9.18 ^a^	− 9.18 ^a^
	Distribution	0.5SD	10.66 ^a^	− 10.66 ^a^
	Linear regression	QoL GHS	9.31	− 6.81
	Linear regression	CD-RISC-10	8.56	− 6.19
	Linear regression	HADS-A	8.78	− 7.05
	Linear regression	HADS-D	8.97	− 6.79
	ROC	QoL-total	5.50	− 4.50
	ROC	CD-RISC-10	5.50	− 4.50
	ROC	HADS-A	5.50	− 4.50
	ROC	HADS-D	5.50	− 4.50

^a^ Mean

## Discussion

A prospective study was performed to assess the MCIDs of RS-SCs using distribution- and anchor-based methods. The findings for RS-SCs were robust as regards the choice of comparator instrument based on the large sample (623 paired RS-SCs scores) and high response rates (84.1%). It demonstrated the convergent validity of the RS-SCs based on the significant correlations with established disease-specific scales (anchors) at baseline. In general, the correlations between RS-SC-10 and established scales were stronger than those between RS-SC-25 and other external indicators, indicating that RS-SC-10 would more likely be of interest to researchers in patients with cancer. Also, it meant that a two-factor structure was better to capture resilience characteristics than the original five-factor one, and the theoretical framework of resilience in cancer patients based on RS-SCs should be revised and redefined in future studies. In addition, we found the significant correlations between changes in RS-SCs and in anchors, providing implications for the design of resilience-related clinical intervention trials [12, 13], especially for sample size calculation; for example, a correlation coefficient of 0.31 between changes in QoL-GHS and in RS-SCs was identified at T1, and as such, 132 paired measurements should be recruited to achieve 90% power and a 0.05 significance level with an anticipated dropout of 20% [37].

Although the determination of MCID remains controversial and researchers have not established consensus and methods to determine the MCID of patient-reported outcome measures, it is important in the validation of clinical instrument studies [38]. In relation to this, two main approaches, distribution- and anchor-based methods, were both performed in this study. Distribution-based methods rely on the distribution of the cohort population and the reliability of the instrument instead of the patients’ perspective. A range of different estimates of the MCID have been proposed, including SEM, 0.5 SD, and MDC_90_, leading to inconsistencies and significantly limited clinical interpretation [39]. As expected, the distribution-based estimates of 0.5 SD and SEM were similar to the anchor-based estimates in the current study. However, the MDC_90_ and MDC_95_ estimates for MCID were much higher compared with other distribution- or anchor-based estimates because the MDC estimates took SD, SEM, and CI into consideration [39]. In addition, we found that the distribution-based estimates of MCID were consistently greater than the anchor-based estimates, indicating a wide distribution of RS-SCs in the cohorts. Anchor-based methods, which take information on patient-reported benefit or deterioration into account, are often preferred to distribution-based methods, based on the comparison of the changes in outcomes of interest with established outcomes of change. However, no consensus has been reached as regards the threshold strength of the correlations between the outcome of interest with other anchors, and patient-reported outcomes recorded before and after interventions are subject to recall bias, which will also affect the strength of the correlations [40]. Therefore, sensitivity and specificity analyses were performed at the individual level (within subject) in our study. The MCID estimations for RS-SC-10 and RS-SC-25 yielded approximately + 2.0 and + 5.5 for improvement and − 1.5 and − 4.5 for deterioration, respectively, indicating that the RS-SCs were more responsive to the worsening status of patients with cancer. For example, a patient’s RS-SC-25 decreasing by − 4.5 or increasing by + 5.5 would suggest that the health state of resilience has clinically changed and would be associated with other psychosocial functions (i.e. anxiety and depression). The same applies for RS-SC-10. We chose anchor-based estimates over distribution-based ones for two reasons. First, distribution-based estimates cannot directly measure MCIDs although they provide indirect and supportive information with regard to significant changes in the instrument; this view is shared by other investigators [41]. Second, the MCIDs estimated by distribution-based approaches are much greater than the mean change identified by anchor-based methods, indicating that distribution-based estimates may overestimate the true MCIDs [38, 39]. At last, RS-SCs may have potential application in resilience-based evaluation (ie, instruments and theoretical development) [40, 43] and intervention for adolescents with cancer with cancer and their caregivers [44–47], for example, the parents of children with cancer, which is receiving enhanced interest in recent cancer research [48, 49]. More research for this vulnerable group are urgently warranted.

### Limitations

The current study has a number of limitations to be noted. First, owing to deteriorating health status or other reasons for loss to follow-up, a possible selection bias may be identified in RS-SCs, which is not obtained for ethical reasons, and may overestimate or underestimate the strength of correlations [50]. Second, there exists no golden standard for defining and measuring resilience in patients with cancer and we had to choose disease-specific and established instruments as anchors. Thus, the findings in the current study, especially those on the differences between improvement and deterioration in RS-SCs, should be interpreted with caution and must be validated in future research. Third, the floor and ceiling effects of outcome measures should also be noted, which could indicate no room for further improvement or deterioration in health status. The current study could not address whether MCIDs for RS-SCs hold true in populations at extremes of the health spectrum (i.e. a baseline RS-SC-10 of 10 or 50).

## Conclusion

The most reliable MCID is around 5 points for RS-SC-25 and 2 points for RS-SC-10. RS-SCs are more responsive to the worsening status of resilience in patients with cancer and these estimates could be useful in future resilience-based intervention trials.

## Supplementary Information

**Additional file 1: Table S1.** Resilience Scale Specific to Cancer (RS-SC, English Version). **S2 Table.** 10-item Resilience Scale Specific to Cancer (RS-SC-10, English Version).

## Data Availability

The datasets used and/or analyzed during the current study are available from the corresponding author on reasonable request.

## Abbreviations

MCID: Minimum Clinical Important Difference
RS-SC-10: 10-item Resilience Scale Specific to Cancer
RS-SC-25: 25-item Resilience Scale Specific to Cancer
EORTC QLQ-C30: European Organization for Research and Treatment of Cancer Quality of Life Questionnaire
CD-RISC: Connor-Davidson Resilience Scale
HADS: Hospital Anxiety and Depression Scale
ALI: Allostatic Load Index
SEM: Standard Error of Measurement
MDC: Minimal Detectable Change
AUC: Area Under Curve
QoL: Quality of Life
GHS: Global Health Status
PF: Physical Function
RF: Role Function
EF: Emotion Function
CF: Cognitive Function
SF: Social Function
Sen: Sensitivity
Spe: Specificity
YI: Youden Index
